# Seasonal Influenza Vaccine Literacy and Hesitancy of Elderly Czechs: An Analysis Using the 5C Model of Psychological Antecedents

**DOI:** 10.3389/ijph.2024.1607626

**Published:** 2024-10-14

**Authors:** Abanoub Riad, Veronika Truksová, Michal Koščík

**Affiliations:** ^1^ Department of Public Health, Faculty of Medicine, Masaryk University, Brno, Czechia; ^2^ Masaryk Centre for Global Health (MCGH), Department of Public Health, Faculty of Medicine, Masaryk University, Brno, Czechia

**Keywords:** aged, Czech Republic, health literacy, influenza, vaccination hesitancy

## Abstract

**Objectives:**

Seasonal influenza vaccination rates among the elderly in the Czech Republic are alarmingly low, making it one of the least vaccinated countries in Europe. This study explored the role of vaccine literacy and insurance coverage on vaccination status.

**Methods:**

An analytical cross-sectional study was conducted in Summer 2023 using a self-administered questionnaire covering vaccine literacy (functional, interactive, and critical skills), negative perceptions towards influenza vaccination, and the 5C model (confidence, complacency, constraints, calculation, and collective responsibility). Individuals aged 55 and older were included in the study. Mediation analyses assessed the indirect effects of insurance coverage on vaccination status.

**Results:**

Significant differences were noted in vaccination rates based on insurance coverage, chronic diseases, regular medication use, and previous COVID-19 and pneumococcal vaccinations. Vaccine literacy, especially interactive and critical skills, was higher among vaccinated individuals. Confidence and collective responsibility were significant promoters, while complacency and constraints were barriers to vaccination. Mediation analyses indicated that negative perceptions, confidence, and collective responsibility significantly mediated the relationship between insurance coverage and vaccination status.

**Conclusion:**

Enhancing vaccine literacy and addressing psychological antecedents are crucial for improving influenza vaccination rates among the elderly. Policy measures should include improving vaccine literacy, building public confidence, and addressing negative perceptions.

## Introduction

According to the World Health Organization (WHO), an influenza pandemic is a significant threat to global health due to its unpredictable nature [1]. Due to age-related decline in immune function (immunosenescence) and the high prevalence of chronic comorbidities, elderly populations are particularly vulnerable to severe illness, complications, and mortality from seasonal influenza [2]. The WHO estimates that seasonal influenza is responsible for 290,000 to 650,000 deaths annually, with 67% of these deaths occurring in individuals aged 65 and older [3].

Vaccination plays a crucial role in mitigating the impact of seasonal influenza, significantly reducing severe illness, hospitalizations, and mortality [4]. However, influenza vaccine coverage rates vary widely across the globe and within the European Union (EU). The recommended vaccination coverage level in Europe is 75% for the elderly population [5]. As of 2021, only Ireland and Denmark met this recommendation, with the EU average at 50.8%. Notably, the Czech Republic fell below half of the EU–27 average, with only 25.4% of its ≥65-year-old population being vaccinated in 2021 [6]. The vaccination crisis in the Czech Republic is not limited to seasonal influenza, but it is empirically observed with declining coverage rates for nearly all pediatric and adults’ vaccines [7].

Vaccine hesitancy, defined as the delay in acceptance or refusal of vaccines despite the availability of vaccination services [8], is a global health threat that affects elderly populations due to factors such as misinformation, fear of side effects, perceived lack of efficacy, and mistrust in healthcare systems [4, 9]. Enhancing vaccine literacy is essential to address these barriers, as it empowers individuals to make informed health decisions [9].

Vaccine literacy extends the concept of health literacy, focusing on the ability to access, process, and understand vaccination information [10–13]. It includes functional (basic skills), interactive (advanced cognitive and social skills), and critical skills (analytical abilities) [14]. Improving vaccine literacy can help overcome vaccine hesitancy by enabling individuals to navigate vaccination information confidently [10].

The 5C model of vaccination psychological antecedents, developed by Betsch et al., includes confidence (trust in vaccines), complacency (low perceived risk of infection), constraints (barriers to vaccination), calculation (weighing pros and cons), and collective responsibility (willingness to protect others) [15]. These antecedents are significantly associated with vaccination intentions and behaviours in various population groups [16–18].

In the Czech Republic, there is a universal statutory health insurance system that covers the entire national population including preventive services, e.g., vaccination. Influenza vaccines are recommended for adults aged 65 and older and for those with specific chronic diseases (chronic cardiovascular disease, chronic kidney disease, and diabetes mellitus), with full insurance coverage for these groups [19]. Likewise, pneumococcal disease vaccines are recommended and covered for the adults aged 65 and older and for those with chronic conditions (asthma, chronic cardiovascular disease, and immune disorder) [19]. Despite growing evidence on the economic and health benefits of age-lowering policies for vaccine recommendation, it remains unclear how expanding the pool of eligible older adults would impact coverage rates beyond alleviating the financial barrier [20–22]. Therefore, research is needed to explore the age-specific implications of insurance coverage and its potential effects on vaccination uptake.

This study aims to assess seasonal influenza vaccine literacy and hesitancy among the elderly population in the Czech Republic. The primary objectives are a) to evaluate vaccine literacy levels and its sociodemographic and anamnestic determinants and b) to evaluate seasonal influenza vaccine hesitancy using the 5C model and its sociodemographic and anamnestic determinants. The secondary objective is to examine the potential effects of insurance coverage on vaccination uptake and explore the implications of expanding vaccination recommendations.

## Methods

### Design

An analytical cross-sectional survey was conducted in Summer 2023 to evaluate seasonal influenza vaccine literacy and perceptions among the elderly in the Czech Republic. The study utilised an online self-administered questionnaire (SAQ) developed and disseminated using KoboToolbox (Kobo Inc., Cambridge, MA, United States, 2023) [23]. The STrengthening the Reporting of OBservational studies in Epidemiology (STROBE) guidelines were followed [24].

### Participants

The target population comprised senior adults in the Czech Republic. Inclusion criteria were: a) aged 55 or older, b) fluent in Czech, c) residing in a private home or elderly/nursing home, and d) having a vaccination status for seasonal influenza. Exclusion criteria included: a) under 55 years of age, and b) non-disclosure of seasonal influenza vaccination history. The decision to include individuals aged 55 and older, rather than limiting the sample to those aged 65 and older, was made to allow for a sub-group analysis. This enabled the comparison of two groups: Group A (aged 55–64) who are not covered by insurance for influenza vaccination, and Group B (aged 65 and older) who are covered by insurance.

### Sample Size

Considering a target population of 3.4 million people aged 55 and older in the Czech Republic, the sample size was calculated using OpenEpi (Dean AG, Atlanta, GA, United States, 2023). Assuming a 50% outcome frequency, 5% error margin, and 95% confidence level, the required sample was 384 respondents [25, 26].

### Data Collection

Collaboration was sought from senior leisure and community organisations, such as Senioři České republiky z.s., Svaz tělesně postižených v České republice z.s., and Universities of the Third Age. The online questionnaire was distributed with a request to share it within respondents’ networks.

### Variables

The SAQ comprised 53 items, including multiple-choice questions and Likert scales, divided into sections: a) sociodemographic characteristics (age, sex, education level, employment status); b) general anamnestic characteristics (BMI, smoking status, chronic diseases, frequently administered medications); c) immunization anamnesis (seasonal influenza, COVID-19, and pneumococcal vaccination status); d) vaccine literacy; e) negative perceptions towards vaccination; and f) psychological antecedents of vaccination.

Vaccine literacy (VL) was measured by eleven items using 4-point Likert scales (1 = never, 2 = rarely, 3 = sometimes, 4 = often) and divided into functional skills (4 items) and interactive and critical skills (7 items). The VL scale developed by Biasio et al. demonstrated satisfactory psychometric properties and was utilised in the present study [27].

Negative perceptions towards influenza vaccination were measured by six items using 5-point Likert scales (1 = strongly disagree to 5 = strongly agree). These items were first used by Gendler et al. and they assessed beliefs about side effects, potential lasting health complications, necessity of vaccination for healthy individuals, perceived immune system robustness, importance of vaccination for older adults with chronic diseases, and the misconception of herd immunity [28].

Psychological antecedents, known as the 5C model, were adapted for this study. Twelve items using 5-point Likert scales (1 = strongly disagree, 2 = disagree, 3 = not sure, 4 = agree, 5 = strongly agree) were employed [15, 29]. Our confirmatory factor analysis indicated acceptable model fit (CFI = 0.967, TLI = 0.952, SRMR = 0.040, RMSEA = 0.059).

The overall scores ranged as follows: functional skills (4–16), interactive and critical skills (7–28), negative perceptions (6–30), confidence (3–15), complacency (1–5), constraints (2–10), calculation (3–15), and collective responsibility (3–15).

A pragmatic approach for translation and cross-cultural adaptation was employed, involving two independent forward translations and an expert panel review to resolve discrepancies [30].

Prior to launching the survey, a pilot phase was conducted with a small group of seniors (*n* = 5) to assess the clarity and comprehension of the questionnaire items. After collecting the first 20 responses, the psychometric properties of the instruments were tested, and a re-validation was performed at the end of the data collection phase.

### Ethics

The study was approved by the Ethics Committee of the Faculty of Medicine, Masaryk University, on 21 March 2023 (reference number 3/2023). The Declaration of Helsinki and GDPR guidelines were followed [31, 32]. Participants provided informed consent digitally and could withdraw at any time without negative consequences. They were not offered any incentives for participation and their identity was kept anonymous throughout the study.

### Analyses

Normal distribution of dependent numerical variables was evaluated using the Shapiro-Wilk test. Descriptive statistics were carried out using frequencies and percentages for qualitative variables, and medians and inter-quartile ranges for numerical variables. Chi-squared test, Fisher’s exact test, Mann-Whitney test, Kruskal-Wallis test, Spearman’s correlation, and multi-variable logistic regression were conducted with a significance level of <0.05. Mediation analyses assessed the indirect effects of insurance coverage on vaccination status through psychological and behavioural mediators. All statistical tests were performed using SPSS 28 (IBM Corp., Armonk, NY, United States, 2023) and Jamovi (The Jamovi Project, Sydney, Australia, 2023) [33, 34].

## Results

### Sample Characteristics

A total of 399 responses were received, of which 15 were excluded for being below 55 years of age, leaving 384 for subsequent analyses. The majority of participants (79.4%) were female, and the median age was 68.5 years, with 67.7% aged 65 years or older. Regarding educational attainment, 1.6% had completed elementary school, 55.2% secondary school, and 43.2% held university degrees. Most participants (60.2%) relied solely on pensions, while 39.8% had additional income sources. The vast majority were permanent residents of their own households (99%), and only 12.2% were smokers.

Approximately 44% of participants reported having at least one chronic disease, with chronic hypertension being the most prevalent (48.5%), followed by thyroid disorders (26.6%), allergies (26.6%), cardiovascular disease (21.3%), type-2 diabetes mellitus (19.5%), and asthma (18.9%). Additionally, 80.7% reported taking medications regularly. Most participants (93.2%) had received at least one dose of a COVID-19 vaccine. Only 27.1% reported having received the pneumococcal vaccine.

Regarding seasonal influenza vaccination status, 62.2% had ever been vaccinated. Among those, 72.8% received a vaccine dose in the last 12 months. The most commonly cited provider was a general practitioner (83.7%), followed by vaccination centres (15.9%) and social/healthcare staff (0.4%). Only 10.9% of those ever vaccinated were infected with seasonal influenza in the same season.

Participants who were covered by insurance (66.9% “of those who were covered” vs. 52.4% “of those who were not covered”; *p* = 0.006), suffering from chronic diseases (70.4% vs. 55.8%; *p* = 0.003), receiving medication regularly (64.8% vs. 51.4%; *p* = 0.032), immunized against COVID-19 (64.2% vs. 34.6%; *p* = 0.003), and immunized against pneumococcal infection (77.9% vs. 56.4%; *p.* < 0.001) had significantly higher rates of seasonal influenza vaccination compared to their counterparts. The remaining sociodemographic and anamnestic characteristics were not significantly associated with seasonal influenza vaccination status (Supplementary Table S1).

### Vaccine Literacy

When asked about their experience with listening to or reading information about vaccines, 71.4% of participants reported encountering unknown words, 54.9% found the texts difficult to understand, 48.7% needed considerable time to comprehend them, and only 27.3% sought help to understand. These four statements constituted the functional skills construct, with a median score of 6 [4–8] points, showing no significant difference between ever-vaccinated and never-vaccinated participants.

Evaluating interactive and critical skills, 70.6% reported consulting more than one source of information, with significant differences between ever-vaccinated (77%) and never-vaccinated (60%) participants. Additionally, 79.9% found the information they were searching for, 73.2% used the information, and 59.4% discussed what they understood with doctors. Furthermore, 78.1% considered the credibility of the sources, 78.6% double-checked the correctness of the information, and 80.7% found useful information to make an informed decision about vaccination. The median score of the interactive and critical skills construct was 20 [14–24], significantly different between ever-vaccinated and never-vaccinated participants (21 vs. 18; *p.* < 0.001, respectively) (Table 1).

**TABLE 1:** Vaccine literacy, negative perceptions, and psychologic antecedents of senior Czechs responding to the Influenza Vaccination Survey, Czech Republic, April–August 2023 (*n* = 384).

Construct	Item	Outcome	Never vaccinated (*n* = 145)	Ever vaccinated (*n* = 239)	Total (*n* = 384)	*Sig.*
Vaccine Literacy: Functional Skills	Did you find words you didn’t know?	Never = 1	44 (30.3%)	66 (27.6%)	110 (28.6%)	0.133
		Rarely = 2	49 (33.8%)	108 (45.2%)	157 (40.9%)	
		Sometimes = 3	45 (31%)	54 (22.6%)	99 (25.8%)	
		Often = 4	7 (4.8%)	11 (4.6%)	18 (4.7%)	
		Median (IQR)	2 (1–3)	2 (1–3)	2 (1–3)	0.492
	Did you find that the texts were difficult to understand?	Never = 1	60 (41.4%)	113 (47.3%)	173 (45.1%)	0.646
		Rarely = 2	56 (38.6%)	79 (33.1%)	135 (35.2%)	
		Sometimes = 3	23 (15.9%)	39 (16.3%)	62 (16.1%)	
		Often = 4	6 (4.1%)	8 (3.3%)	14 (3.6%)	
		Median (IQR)	2 (1–2)	2 (1–2)	2 (1–2)	0.370
	Did you need much time to understand them?	Never = 1	72 (49.7%)	125 (52.3%)	197 (51.3%)	0.391
		Rarely = 2	47 (32.4%)	71 (29.7%)	118 (30.7%)	
		Sometimes = 3	24 (16.6%)	33 (13.8%)	57 (14.8%)	
		Often = 4	2 (1.4%)	10 (4.2%)	12 (3.1%)	
		Median (IQR)	2 (1–2)	1 (1–2)	1 (1–2)	0.768
	Did you or would you need someone to help you understand them?	Never = 1	108 (74.5%)	171 (71.5%)	279 (72.7%)	0.749
		Rarely = 2	22 (15.2%)	42 (17.6%)	64 (16.7%)	
		Sometimes = 3	11 (7.6%)	22 (9.2%)	33 (8.6%)	
		Often = 4	4 (2.8%)	4 (1.7%)	8 (2.1%)	
		Median (IQR)	1 (1–2)	1 (1–2)	1 (1–2)	0.579
	Overall Score of Functional Skills	Median (IQR)	7 (4–9)	6 (4–8)	6 (4–8)	0.664
Vaccine Literacy: Interactive and Critical Skills	Have you consulted more than one source of information?	Never = 1	58 (40%)	55 (23%)	113 (29.4%)	**0.001**
		Rarely = 2	31 (21.4%)	66 (27.6%)	97 (25.3%)	
		Sometimes = 3	40 (27.6%)	66 (27.6%)	106 (27.6%)	
		Often = 4	16 (11%)	52 (21.8%)	68 (17.7%)	
		Median (IQR)	2 (1–3)	2 (2–3)	2 (1–3)	**<0.001**
	Did you find the information you were looking for?	Never = 1	43 (29.7%)	34 (14.2%)	77 (20.1%)	**<0.001**
		Rarely = 2	20 (13.8%)	23 (9.6%)	43 (11.2%)	
		Sometimes = 3	39 (26.9%)	54 (22.6%)	93 (24.2%)	
		Often = 4	43 (29.7%)	128 (53.6%)	171 (44.5%)	
		Median (IQR)	3 (1–4)	4 (3–4)	3 (2–4)	**<0.001**
	Have you had the opportunity to use the information?	Never = 1	62 (42.8%)	41 (17.2%)	103 (26.8%)	**<0.001**
		Rarely = 2	28 (19.3%)	34 (14.2%)	62 (16.1%)	
		Sometimes = 3	32 (22.1%)	67 (28%)	99 (25.8%)	
		Often = 4	23 (15.9%)	97 (40.6%)	120 (31.3%)	
		Median (IQR)	2 (1–3)	3 (2–4)	3 (1–4)	**<0.001**
	Did you discuss what you understood about vaccinations with your doctor or other people?	Never = 1	84 (57.9%)	72 (30.1%)	156 (40.6%)	**<0.001**
		Rarely = 2	28 (19.3%)	70 (29.3%)	98 (25.5%)	
		Sometimes = 3	24 (16.6%)	57 (23.8%)	81 (21.1%)	
		Often = 4	9 (6.2%)	40 (16.7%)	49 (12.8%)	
		Median (IQR)	1 (1–2)	2 (1–3)	2 (1–3)	**<0.001**
	Have you considered the credibility of the sources?	Never = 1	48 (33.1%)	36 (15.1%)	84 (21.9%)	**<0.001**
		Rarely = 2	11 (7.6%)	33 (13.8%)	44 (11.5%)	
		Sometimes = 3	35 (24.1%)	55 (23%)	90 (23.4%)	
		Often = 4	51 (35.2%)	115 (48.1%)	166 (43.2%)	
		Median (IQR)	3 (1–4)	3 (2–4)	3 (2–4)	**0.001**
	Did you check whether the information was correct?	Never = 1	48 (33.1%)	34 (14.2%)	82 (21.4%)	**<0.001**
		Rarely = 2	17 (11.7%)	33 (13.8%)	50 (13%)	
		Sometimes = 3	30 (20.7%)	59 (24.7%)	89 (23.2%)	
		Often = 4	50 (34.5%)	113 (47.3%)	163 (42.4%)	
		Median (IQR)	3 (1–4)	3 (2–4)	3 (2–4)	**<0.001**
	Did you find any useful information to make a decision on whether or not to get vaccinated?	Never = 1	40 (27.6%)	34 (14.2%)	74 (19.3%)	**<0.001**
		Rarely = 2	11 (7.6%)	20 (8.4%)	31 (8.1%)	
		Sometimes = 3	46 (31.7%)	50 (20.9%)	96 (25%)	
		Often = 4	48 (33.1%)	135 (56.5%)	183 (47.7%)	
		Median (IQR)	3 (1–4)	4 (3–4)	3 (2–4)	**<0.001**
	Overall Score of Interactive and Critical Skills	Median (IQR)	18 (10–22)	21 (17–25)	20 (14–24)	**<0.001**
Negative Perceptions	The seasonal influenza shot causes serious side effects	Strongly Disagree = 1	10 (6.9%)	60 (25.1%)	70 (18.2%)	**<0.001**
		Disagree = 2	32 (22.1%)	117 (49%)	149 (38.8%)	
		Not Sure = 3	78 (53.8%)	50 (20.9%)	128 (33.3%)	
		Agree = 4	19 (13.1%)	8 (3.3%)	27 (7%)	
		Strongly Agree = 5	6 (4.1%)	4 (1.7%)	10 (2.6%)	
		Median (IQR)	3 (2–3)	2 (1–3)	2 (2–3)	**<0.001**
	The seasonal influenza vaccine can cause permanent health problems	Strongly Disagree = 1	10 (6.9%)	51 (21.3%)	61 (15.9%)	**<0.001**
		Disagree = 2	43 (29.7%)	116 (48.5%)	159 (41.4%)	
		Not Sure = 3	63 (43.4%)	61 (25.5%)	124 (32.3%)	
		Agree = 4	22 (15.2%)	11 (4.6%)	33 (8.6%)	
		Strongly Agree = 5	7 (4.8%)	0 (0%)	7 (1.8%)	
		Median (IQR)	3 (2–3)	2 (2–3)	2 (2–3)	**<0.001**
	I do not need to get vaccinated against seasonal influenza if I am in good health	Strongly Disagree = 1	4 (2.8%)	60 (25.1%)	64 (16.7%)	**<0.001**
		Disagree = 2	3 (2.1%)	101 (42.3%)	104 (27.1%)	
		Not Sure = 3	40 (27.6%)	42 (17.6%)	82 (21.4%)	
		Agree = 4	60 (41.4%)	26 (10.9%)	86 (22.4%)	
		Strongly Agree = 5	38 (26.2%)	10 (4.2%)	48 (12.5%)	
		Median (IQR)	4 (3–5)	2 (1–3)	3 (2–4)	**<0.001**
	I do not need to get a seasonal influenza shot because I have a strong immune system and there is a good chance that I will have a mild course	Strongly Disagree = 1	5 (3.4%)	65 (27.2%)	70 (18.2%)	**<0.001**
		Disagree = 2	7 (4.8%)	90 (37.7%)	97 (25.3%)	
		Not Sure = 3	49 (33.8%)	60 (25.1%)	109 (28.4%)	
		Agree = 4	52 (35.9%)	17 (7.1%)	69 (18%)	
		Strongly Agree = 5	32 (22.1%)	7 (2.9%)	39 (10.2%)	
		Median (IQR)	4 (3–4)	2 (1–3)	3 (2–4)	**<0.001**
	Only older people with more serious chronic diseases should be vaccinated	Strongly Disagree = 1	6 (4.1%)	57 (23.8%)	63 (16.4%)	**<0.001**
		Disagree = 2	17 (11.7%)	78 (32.6%)	95 (24.7%)	
		Not Sure = 3	47 (32.4%)	46 (19.2%)	93 (24.2%)	
		Agree = 4	45 (31%)	35 (14.6%)	80 (20.8%)	
		Strongly Agree = 5	30 (20.7%)	23 (9.6%)	53 (13.8%)	
		Median (IQR)	4 (3–5)	2 (2–3)	3 (2–4)	**<0.001**
	When everyone else around me is vaccinated, I don’t have to get vaccinated	Strongly Disagree = 1	28 (19.3%)	83 (34.7%)	111 (28.9%)	**<0.001**
		Disagree = 2	38 (26.2%)	114 (47.7%)	152 (39.6%)	
		Not Sure = 3	59 (40.7%)	26 (10.9%)	85 (22.1%)	
		Agree = 4	11 (7.6%)	10 (4.2%)	21 (5.5%)	
		Strongly Agree = 5	9 (6.2%)	6 (2.5%)	15 (3.9%)	
		Median (IQR)	3 (2–3)	2 (1–2)	2 (1–3)	**<0.001**
	Overall Score of Negative Perceptions	Median (IQR)	19 (17–21)	13 (10–16)	16 (12–19)	**<0.001**
Confidence	I am completely confident that seasonal influenza vaccines are safe	Strongly Disagree = 1	9 (6.2%)	3 (1.3%)	12 (3.1%)	**<0.001**
		Disagree = 2	26 (17.9%)	5 (2.1%)	31 (8.1%)	
		Not Sure = 3	68 (46.9%)	67 (28%)	135 (35.2%)	
		Agree = 4	34 (23.4%)	118 (49.4%)	152 (39.6%)	
		Strongly Agree = 5	8 (5.5%)	46 (19.2%)	54 (14.1%)	
		Median (IQR)	3 (3–4)	4 (3–4)	4 (3–4)	**<0.001**
	I am completely confident that seasonal influenza vaccines are effective	Strongly Disagree = 1	7 (4.8%)	2 (0.8%)	9 (2.3%)	**<0.001**
		Disagree = 2	24 (16.6%)	6 (2.5%)	30 (7.8%)	
		Not Sure = 3	65 (44.8%)	48 (20.1%)	113 (29.4%)	
		Agree = 4	40 (27.6%)	133 (55.6%)	173 (45.1%)	
		Strongly Agree = 5	9 (6.2%)	50 (20.9%)	59 (15.4%)	
		Median (IQR)	3 (3–4)	4 (4–4)	4 (3–4)	**<0.001**
	Regarding seasonal influenza, I am confident that public authorities decide in the best interest of the community	Strongly Disagree = 1	17 (11.7%)	9 (3.8%)	26 (6.8%)	**<0.001**
		Disagree = 2	34 (23.4%)	30 (12.6%)	64 (16.7%)	
		Not Sure = 3	69 (47.6%)	92 (38.5%)	161 (41.9%)	
		Agree = 4	21 (14.5%)	79 (33.1%)	100 (26%)	
		Strongly Agree = 5	4 (2.8%)	29 (12.1%)	33 (8.6%)	
		Median (IQR)	3 (2–3)	3 (3–4)	3 (3–4)	**<0.001**
Complacency	Repeating vaccination against seasonal influenza for people at higher risk of influenza complications is unnecessary	Strongly Disagree = 1	11 (7.6%)	52 (21.8%)	63 (16.4%)	**<0.001**
		Disagree = 2	42 (29%)	107 (44.8%)	149 (38.8%)	
		Not Sure = 3	71 (49%)	61 (25.5%)	132 (34.4%)	
		Agree = 4	13 (9%)	15 (6.3%)	28 (7.3%)	
		Strongly Agree = 5	8 (5.5%)	4 (1.7%)	12 (3.1%)	
		Median (IQR)	3 (2–3)	2 (2–3)	2 (2–3)	**<0.001**
Constraints	For me, it is inconvenient to be vaccinated against seasonal influenza every year	Strongly Disagree = 1	10 (6.9%)	73 (30.5%)	83 (%21.6)	**<0.001**
		Disagree = 2	21 (14.5%)	99 (41.4%)	120 (31.3%)	
		Not Sure = 3	50 (34.5%)	33 (13.8%)	83 (21.6%)	
		Agree = 4	39 (26.9%)	23 (9.6%)	62 (16.1%)	
		Strongly Agree = 5	25 (17.2%)	11 (4.6%)	36 (9.4%)	
		Median (IQR)	3 (3–4)	2 (1–3)	2 (2–4)	**<0.001**
	Visiting the doctor makes me feel uncomfortable; this keeps me from being vaccinated against seasonal influenza	Strongly Disagree = 1	29 (20%)	105 (%)	134 (%)	**<0.001**
		Disagree = 2	42 (29%)	99 (%)	141 (%)	
		Not Sure = 3	48 (33.1%)	27 (%)	75 (%)	
		Agree = 4	15 (10.3%)	7 (%)	22 (%)	
		Strongly Agree = 5	11 (7.6%)	1 (%)	12 (%)	
		Median (IQR)	3 (2–3)	2 (1–2)	2 (1–3)	**<0.001**
Calculation	When I think about being vaccinated against seasonal influenza, I weigh its benefits and risks to make the best decision possible	Strongly Disagree = 1	5 (3.4%)	7 (2.9%)	12 (3.1%)	**0.006**
		Disagree = 2	4 (2.8%)	23 (9.6%)	27 (7%)	
		Not Sure = 3	47 (32.4%)	45 (18.8%)	92 (24%)	
		Agree = 4	62 (42.8%)	116 (48.5%)	178 (46.4%)	
		Strongly Agree = 5	27 (18.6%)	48 (20.1%)	75 (19.5%)	
		Median (IQR)	4 (3–4)	4 (3–4)	4 (3–4)	0.468
	I closely consider whether seasonal influenza vaccine is useful for me	Strongly Disagree = 1	4 (2.8%)	9 (3.8%)	13 (3.4%)	**0.008**
		Disagree = 2	5 (3.4%)	26 (10.9%)	31 (8.1%)	
		Not Sure = 3	42 (29%)	51 (21.3%)	93 (24.2%)	
		Agree = 4	57 (39.3%)	113 (47.3%)	170 (44.3%)	
		Strongly Agree = 5	37 (25.5%)	40 (16.7%)	77 (20.1%)	
		Median (IQR)	4 (3–5)	4 (3–4)	4 (3–4)	0.115
	It is important for me to fully understand the topic of vaccination before I get my vaccination	Strongly Disagree = 1	4 (2.8%)	5 (2.1%)	9 (2.3%)	0.172
		Disagree = 2	3 (2.1%)	11 (4.6%)	14 (3.6%)	
		Not Sure = 3	30 (20.7%)	50 (20.9%)	80 (20.8%)	
		Agree = 4	62 (42.8%)	121 (50.6%)	183 (47.7%)	
		Strongly Agree = 5	46 (31.7%)	52 (21.8%)	98 (25.5%)	
		Median (IQR)	4 (3–5)	4 (3–4)	4 (3–5)	0.106
Collective Responsibility	Like everyone else, I must be vaccinated against seasonal influenza	Strongly Disagree = 1	46 (31.7%)	27 (11.3%)	73 (19%)	**<0.001**
		Disagree = 2	60 (41.4%)	94 (39.3%)	154 (40.1%)	
		Not Sure = 3	36 (24.8%)	64 (26.8%)	100 (26%)	
		Agree = 4	2 (1.4%)	38 (15.9%)	40 (10.4%)	
		Strongly Agree = 5	1 (0.7%)	16 (6.7%)	17 (4.4%)	
		Median (IQR)	2 (1–3)	2 (2–3)	2 (2–3)	**<0.001**
	Being vaccinated against seasonal influenza also protects other people at higher risk of influenza complications and with weaker immune system	Strongly Disagree = 1	20 (13.8%)	4 (1.7%)	24 (6.3%)	**<0.001**
		Disagree = 2	22 (15.2%)	17 (7.1%)	39 (10.2%)	
		Not Sure = 3	63 (43.4%)	35 (14.6%)	98 (25.5%)	
		Agree = 4	31 (21.4%)	103 (43.1%)	134 (34.9%)	
		Strongly Agree = 5	9 (6.2%)	80 (33.5%)	89 (23.2%)	
		Median (IQR)	3 (2–4)	4 (4–5)	4 (3–4)	**<0.001**
	Vaccination is a collective action to prevent the spread of diseases	Strongly Disagree = 1	5 (3.4%)	0 (0%)	5 (1.3%)	**<0.001**
		Disagree = 2	15 (10.3%)	9 (3.8%)	24 (6.3%)	
		Not Sure = 3	47 (32.4%)	24 (10%)	71 (18.5%)	
		Agree = 4	56 (38.6%)	122 (51%)	178 (46.4%)	
		Strongly Agree = 5	22 (15.2%)	84 (35.1%)	106 (27.6%)	
		Median (IQR)	4 (3–4)	4 (4–5)	4 (3–5)	**<0.001**
5-C Scores	Confidence	Median (IQR)	9 (7–10)	11 (10–12)	11 (9–12)	**<0.001**
	Complacency	Median (IQR)	3 (2–3)	2 (2–3)	2 (2–3)	**<0.001**
	Constraints	Median (IQR)	6 (5–7)	4 (2–5)	4 (3–6)	**<0.001**
	Calculation	Median (IQR)	12 (10–13)	12 (10–12)	12 (10–13)	0.319
	Collective Responsibility	Median (IQR)	9 (7–10)	11 (10–12)	10 (9–11)	**<0.001**

Chi-squared test (*χ* ^*2*^), Fisher’s exact test, and Mann-Whitney test (*U*) were used with a significance level < 0.05.

Bold font is for statistically significant values *p* < 0.05.

### Negative Perceptions

Negative perceptions towards seasonal influenza vaccination were significantly more common among never-vaccinated participants. Specifically, 9.6% believed that the vaccine could cause serious side effects, with a significant difference between never-vaccinated (17.2%) and ever-vaccinated (5%) participants. Similarly, 10.4% believed that the vaccine could cause lasting health problems (20% vs. 4.6%), 34.9% thought vaccination was unnecessary due to good health (67.6% vs. 15.1%), 28.1% trusted their immune system (57.9% vs. 10%), 34.6% believed vaccination should be limited to older adults with chronic diseases (51.7% vs. 24.3%), and 9.4% thought they did not need vaccination if others were immunized (13.8% vs. 6.7%). The median overall score of negative perceptions was 16 [12–19], significantly different between never-vaccinated and ever-vaccinated participants (19 vs. 13; *p.* < 0.001, respectively) (Table 1).

### Psychological Antecedents (5-C)

More than half of the participants (53.6%) were confident that influenza vaccines were safe, with significant (*p.* < 0.001) differences between ever-vaccinated (68.6%) and never-vaccinated (29%) participants. Similarly, 60.4% were confident that vaccines were effective, and 34.6% were confident that public authorities made decisions in the community’s best interest. The median overall score of the confidence construct was 11 [9–12], significantly higher among ever-vaccinated participants (11 vs. 9; *p.* < 0.001).

Conversely, never-vaccinated participants were more agreeable with the statement “repetitive vaccination is unnecessary” than the ever-vaccinated ones (14.5% vs. 7.9%, respectively). Additionally, 25.5% reported that annual vaccination was inconvenient (never-vaccinated: 44.1% vs. ever-vaccinated: 14.2%), and 8.9% indicated that visiting a doctor was a barrier due to discomfort (never-vaccinated: 17.9% vs. ever-vaccinated: 3.3%). The median overall score of the constraints construct was 4 [3–6], significantly higher among never-vaccinated participants (6 vs. 4; *p*. < 0.001).

Regarding the calculation construct, 65.9% reported weighing the benefits against the risks before vaccination, 64.3% considered the vaccine’s usefulness, and 73.2% acknowledged the importance of understanding vaccination before getting vaccinated. No significant differences were found between never-vaccinated and ever-vaccinated participants.

For the collective responsibility construct, 14.8% agreed that everyone must be vaccinated against influenza, with significant (*p*. < 0.001) differences between ever-vaccinated (22.6%) and never-vaccinated (2.1%) participants. Additionally, 58.1% agreed that vaccination protects others at higher risk, and 74.0% acknowledged vaccination as a collective action to prevent disease spread. The median overall score of the collective responsibility construct was 10 [9–11], significantly higher among ever-vaccinated participants (11 vs. 9; *p*. < 0.001) (Table 1).

### Determinants of Literacy, Perceptions and Antecedents

Male participants had significantly higher functional skills (7 [5–9] vs. 6 [4–8]) and lower calculation scores (11 [9–12] vs. 12 [10–13]) than females. Participants with insurance coverage showed lower negative perceptions (15 [11.25–19] vs. 17 [12.25–19]) and higher confidence (11 [9–12] vs. 10 [8–11]), with lower complacency (2 [2–3] vs. 3 [2–3]) and higher collective responsibility (10 [9–12] vs. 9 [8–11]).

Participants with chronic diseases scored higher in interactive and critical skills (21 [17–24.5] vs. 19 [13–23]), lower in negative perceptions (14 [11–18] vs. 16 [12–20]), and higher in confidence (11 [9–12] vs. 10 [9–12]), with lower complacency (2 [2–3] vs. 3 [2–3]) and constraints (4 [2.5–6] vs. 5 [4–6]), and higher collective responsibility (11 [9–12] vs. 10 [8–11]). Participants on medications showed higher functional skills (7 [5–9] vs. 5 [4–8]), lower negative perceptions (16 [12–19] vs. 17 [12.75–20]), lower constraints (4 [3–6] vs. 5 [3–7]), and higher collective responsibility (10 [9–12] vs. 9.5 [7.75–11]).

Participants vaccinated against COVID-19 had lower negative perceptions (15.5 [12–19] vs. 20.5 [18–23]) and higher confidence (11 [9–12] vs. 7 [4.75–9]), with lower complacency (2 [2–3] vs. 3 [2.75–3]) and constraints (4 [3–6] vs. 6.5 [5–8]), and higher collective responsibility (10 [9–12] vs. 7 [5–9]). Participants vaccinated against pneumococcal infection had lower negative perceptions (13.5 [10–17] vs. 16 [12–20]) and higher confidence (11 [10–12] vs. 10 [9–12]), with lower complacency (2 [2–3] vs. 2 [2–3]) and constraints (4 [2–5] vs. 5 [4–6]), and higher collective responsibility (11 [10–12] vs. 10 [8–11]).

The rest of the sociodemographic and anamnestic characteristics, e.g., smoking status and BMI, were not statistically significant for literacy, negative perceptions or psychological antecedents constructs (Table 2).

**TABLE 2:** Sociodemographic and anamnestic determinants of vaccine literacy, negative perceptions and psychologic antecedents among senior Czechs responding to the Influenza Vaccination Survey, Czech Republic, April–August 2023 (*n* = 384).

Variable	Outcome	Functional Skills (Range: 4–16)	*Sig.*	Interactive/Critical Skills (Range: 7–28)	*Sig.*	Negative Perceptions (Range: 6–30)	*Sig.*	Confidence (Range: 3–15)	*Sig.*
Sex	Female	6 (4–8)	**0.023**	20 (15–24)	0.231	16 (12–19)	0.977	11 (9–12)	0.590
	Male	7 (5–9)		19 (12–23)		16 (13–18)		10 (9–12)	
Covered?	No	7 (5–9)	0.084	20 (13.25–23)	0.271	17 (12.25–19)	**0.020**	10 (8–11)	**0.002**
	Yes	6 (4–8)		21 (14.25–24)		15 (11.25–19)		11 (9–12)	
Education	Elementary	9.5 (4.75–12)	**0.020**	21 (18.5–23.75)	0.652	11.5 (10.5–22.5)	0.871	11 (5.25–12.75)	0.978
	Secondary	7 (5–9)		20 (14–24)		16 (12–19)		11 (9–12)	
	University	6 (4–8)		20 (13–24)		16 (11.75–19)		11 (9–12)	
Employment	Pension Only	7 (5–9)	0.062	21 (14–24)	0.559	16 (12–19)	0.998	11 (9–12)	0.211
	Pension + Additional Sources	6 (4–8)		20 (14–23)		16 (12–19)		10 (8.5–12)	
Smoking	No	6 (4–8)	0.455	20 (14.5–24)	0.329	16 (12–19)	0.193	11 (9–12)	0.197
	Yes	7 (5–9)		19 (14–23)		17 (12–20)		10 (8–11)	
BMI Level	Normal	7 (4–8)	0.929	20 (14–24)	0.567	16 (12–20)	0.817	10 (9–12)	0.670
	Overweight	6 (4–8)		21 (14–24)		15 (12–19)		11 (9–12)	
	Obese	6 (5–9)		19 (12–24)		16 (12–18)		10 (9–12)	
	Extremely Obese	6 (4.25–9)		20.5 (17.25–23.75)		16.5 (13.25–19)		10 (9–12)	
Chronic Diseases	No	6 (4–8)	**0.028**	19 (13–23)	**0.006**	16 (12–20)	**<0.001**	10 (9–12)	**0.035**
	Yes	7 (5–9)		21 (17–24.5)		14 (11–18)		11 (9–12)	
Medications	No	5 (4–8)	**0.015**	19.5 (14–24)	0.694	17 (12.75–20)	**0.018**	10 (8–11.25)	0.112
	Yes	7 (5–9)		20 (14–24)		16 (12–19)		11 (9–12)	
COVID–19 Vaccine	No	7 (4,75–10)	0.574	20.5 (16.25–24)	0.597	20.5 (18–23)	**<0.001**	7 (4.75–9)	**<0.001**
	Yes	6 (4–8)		20 (14–24)		15.5 (12–19)		11 (9–12)	
COVID–19 Vaccine Doses	Primer Doses Only	7 (5–10)	0.124	19 (14–23)	0.636	19 (14–21)	**<0.001**	10 (8–11)	**0.002**
	Primer + 1 Booster	7 (4–9)		21 (14–25)		16 (12–18)		11 (9–12)	
	Primer + 2 Boosters	6 (4–8)		20 (14–24)		14.5 (10.25–18)		11 (9–12)	
Pneumococcal Vaccine	No	6 (4–8)	0.234	20 (14–23.75)	0.060	16 (12–20)	**<0.001**	10 (9–12)	**<0.001**
	Yes	7 (5–9)		21 (16–24.75)		13.5 (10–17)		11 (10–12)	

Variable	Outcome	Complacency (Range: 1–5)	*Sig.*	Constraints (Range: 2–10)	*Sig.*	Calculation (Range: 3–15)	*Sig.*	Collective Responsibility (Range: 3–15)	*Sig.*
Sex	Female	2 (2–3)	0.891	4 (3–6)	0.513	12 (10–13)	**0.016**	10 (9–11)	0.403
	Male	2 (2–3)		4 (3–6)		11 (9–12)		10 (8–12)	
Covered?	No	3 (2–3)	**0.045**	5 (4–6)	0.085	12 (10–13)	0.142	9 (8–11)	**0.008**
	Yes	2 (2–3)		4 (3–6)		12 (9–12)		10 (9–12)	
Education	Elementary	2 (1.75–2.75)	0.752	3.5 (2.75–4.75)	0.446	12 (10.5–15)	0.685	11 (6.75–12.5)	0.800
	Secondary	2 (2–3)		5 (3–6)		12 (10–12)		10 (9–11)	
	University	2 (2–3)		4 (3–6)		12 (9–13)		10 (8–12)	
Employment	Pension Only	2 (2–3)	0.836	4 (3–6)	0.330	12 (10–12)	0.492	10 (9–12)	**0.022**
	Pension + Additional Sources	2 (2–3)		5 (3–6)		12 (9.5–13)		10 (8–11)	
Smoking	No	2 (2–3)	0.985	4 (3–6)	0.449	12 (10–13)	0.207	10 (9–11)	0.598
	Yes	2 (2–3)		5 (4–6)		12 (10–12)		10 (8–12)	
BMI Level	Normal	2 (2–3)	0.388	4 (3–6)	0.929	12 (9–12)	0.538	10 (8.5–12)	0.928
	Overweight	2 (2–3)		4 (3–6)		12 (10–13)		10 (9–11)	
	Obese	3 (2–3)		4 (3–6)		11.5 (9–12)		10 (8–12)	
	Extremely Obese	2 (2–3)		4.5 (3–6)		12 (10–13)		10 (9–11)	
Chronic Diseases	No	3 (2–3)	**0.002**	5 (4–6)	**0.006**	12 (9–13)	0.609	10 (8–11)	**<0.001**
	Yes	2 (2–3)		4 (2.5–6)		12 (10–13)		11 (9–12)	
Medications	No	2 (2–3)	0.590	5 (3–7)	**0.027**	12 (10.75–13)	0.270	9.5 (7.75–11)	**0.009**
	Yes	2 (2–3)		4 (3–6)		12 (9–13)		10 (9–12)	
COVID–19 Vaccine	No	3 (2.75–3)	**0.006**	6.5 (5–8)	**<0.001**	12 (9.75–14.25)	0.307	7 (5–9)	**<0.001**
	Yes	2 (2–3)		4 (3–6)		12 (10–13)		10 (9–12)	
COVID–19 Vaccine Doses	Primer Doses Only	3 (2–3)	**0.014**	5 (4–7)	**<0.001**	12 (9–13)	0.196	9 (8–10)	**<0.001**
	Primer + 1 Booster	2 (2–3)		5 (4–6)		12 (9–12)		10 (8–11)	
	Primer + 2 Boosters	2 (2–3)		4 (3–6)		12 (10–13)		10 (9–12)	
Pneumococcal Vaccine	No	2 (2–3)	**0.017**	5 (4–6)	**<0.001**	12 (9.25–13)	0.913	10 (8–11)	**<0.001**
	Yes	2 (2–3)		4 (2–5)		12 (10–13)		11 (10–12)	

Kruskal-Wallis test (*H*) and Mann–Whitney test (*U*) were used with a significance level < 0.05.

Bold font is for statistically significant values *p* < 0.05.

### Correlation Between Literacy, Perceptions and Antecedents

Non-parametric correlation analysis revealed that negative perceptions were inversely correlated with interactive and critical skills (rho = −0.238) and moderately with confidence (rho = −0.557) and collective responsibility (rho = −0.543). Negative perceptions were directly correlated with complacency (rho = 0.419) and constraints (rho = 0.637).

Interactive and critical skills were directly correlated with confidence (rho = 0.218), calculation (rho = 0.334), and collective responsibility (rho = 0.289), and inversely correlated with complacency (rho = −0.194) and constraints (rho = −0.205).

Among psychological antecedents, calculation was not correlated with any other antecedent. Confidence was inversely correlated with complacency (rho = −0.347) and constraints (rho = −0.501), but directly correlated with collective responsibility (rho = 0.618). Collective responsibility was inversely correlated with complacency (rho = −0.434) and constraints (rho = −0.471) (Table 3).

**TABLE 3:** Non-parametric correlation between Vaccine Literacy, Perceptions, and Psychologic Antecedents among senior Czechs responding to the Influenza Vaccination Survey, Czech Republic, April–August 2023 (*n* = 384).

		Functional Skills	Interactive/critical skills	Negative Perceptions	Confidence	Complacency	Constraints	Calculation	Collective responsibility
Functional Skills	*ρ*	1.000							
	*Sig.*	N/A							
Interactive/Critical Skills	*ρ*	0.011	1.000						
	*Sig.*	0.824	N/A						
Negative Perceptions	*ρ*	0.121	−0.238	1.000					
	*Sig.*	**0.017**	**<0.001**	N/A					
Confidence	*ρ*	−0.154	0.218	−0.557	1.000				
	*Sig.*	**0.003**	**<0.001**	**<0.001**	N/A				
Complacency	*ρ*	0.137	−0.194	0.419	−0.347	1.000			
	*Sig.*	**0.007**	**<0.001**	**<0.001**	**<0.001**	N/A			
Constraints	*ρ*	0.173	−0.205	0.637	−0.501	0.355	1.000		
	*Sig.*	**<0.001**	**<0.001**	**<0.001**	**<0.001**	**<0.001**	N/A		
Calculation	*ρ*	−0.093	0.334	0.071	−0.023	−0.060	0.017	1.000	
	*Sig.*	0.068	**<0.001**	0.167	0.655	0.238	0.733	N/A	
Collective Responsibility	*ρ*	−0.071	0.289	−0.543	0.618	−0.434	−0.471	0.059	1.000
	*Sig.*	0.168	**<0.001**	**<0.001**	**<0.001**	**<0.001**	**<0.001**	0.246	N/A

Bold font is for statistically significant values *p* < 0.05.

### Regression Analysis of Vaccination Determinants

Multivariable logistic regression (MLR) indicated that insurance coverage was associated with higher odds of seasonal influenza vaccination (AOR: 2.40 [95% CI: 1.24–4.63]). Similarly, additional income sources (AOR: 2.33 [95% CI: 1.22–4.43]), chronic diseases (AOR: 1.56 [95% CI: 0.95–2.54]), COVID-19 vaccination (AOR: 3.44 [95% CI: 1.39–8.49]), and pneumococcal infection (AOR: 2.58 [95% CI: 1.44–4.62]) were linked to increased odds of vaccination.

Five MLR models for each psychological antecedent controlled for all sociodemographic and anamnestic variables. Models for confidence and collective responsibility showed higher odds of vaccination (AOR: 1.60 [95% CI: 1.40–1.82] and AOR: 1.80 [95% CI: 1.54–2.09], respectively). Conversely, complacency and constraints were associated with lower odds (AOR: 0.58 [95% CI: 0.45–0.75] and AOR: 0.56 [95% CI: 0.48–0.65], respectively) (Table 4).

**TABLE 4:** Multivariable logistic regression of sociodemographic and anamnestic determinants and psychologic antecedents of influenza vaccination among senior Czechs responding to the Influenza Vaccination Survey, Czech Republic, April–August 2023 (*n* = 384).

Correlate	SE	AOR (95% CI)	*Sig.*
Gender: Male *vs.* Female	0.289	1.29 (0.73–2.27)	0.379
Coverage: Covered *vs.* Not Covered	0.336	2.40 (1.24–4.63)	**0.009**
Education: Secondary *vs.* Elementary	0.959	0.45 (0.07–2.96)	0.408
Education: University *vs.* Elementary	0.965	0.46 (0.07–3.07)	0.426
Income: Pension and Additional Sources *vs.* Pension Only	0.329	2.33 (1.22–4.43)	**0.010**
BMI Level: Overweight *vs.* Normal	0.263	0.97 (0.58–1.61)	0.891
BMI Level: Obese *vs.* Normal	0.356	1.06 (0.53–2.12)	0.876
BMI Level: Extremely Obese *vs.* Normal	0.448	1.14 (0.47–2.75)	0.769
Smoking: Smoker *vs.* Non-smoker	0.350	1.10 (0.56–2.19)	0.779
Chronic Diseases: Yes *vs.* No	0.249	1.56 (0.95–2.54)	0.077
Medications: Yes *vs.* No	0.312	1.07 (0.58–1.97)	0.828
COVID-19 Vaccination: Vaccinated *vs.* Non-vaccinated	0.461	3.44 (1.39–8.49)	**0.007**
Pneumococcal Vaccination: Vaccinated *vs.* Non-vaccinated	0.297	2.58 (1.44–4.62)	**0.001**

Model	Psychologic Antecedent	Nagelkerke R Square	SE	AOR (95% CI)	*Sig.*
1	Confidence	33%	0.066	1.60 (1.40–1.82)	**<0.001**
2	Complacency	19.3%	0.131	0.58 (0.45–0.75)	**<0.001**
3	Constraints	36.2%	0.077	0.56 (0.48–0.65)	**<0.001**
4	Calculation	13.6%	0.047	0.97 (0.89–1.07)	0.578
5	Collective Responsibility	38.3%	0.078	1.80 (1.54–2.09)	**<0.001**

Each psychologic antecedent model was adjusted for gender, health coverage (age group), education level, income, BMI level, smoking, chronic disease, medication, COVID-19 vaccine and pneumococcal vaccine.

Bold font is for statistically significant values *p* < 0.05.

### Insurance Coverage

Mediation analysis aimed to explore the psychological and behavioral mechanisms through which insurance coverage influences seasonal influenza vaccination status. This understanding can identify interventions to complement the suggested policy of lowering the insurance age limit, highlighting additional steps to improve vaccination rates.

**FIGURE 1:**
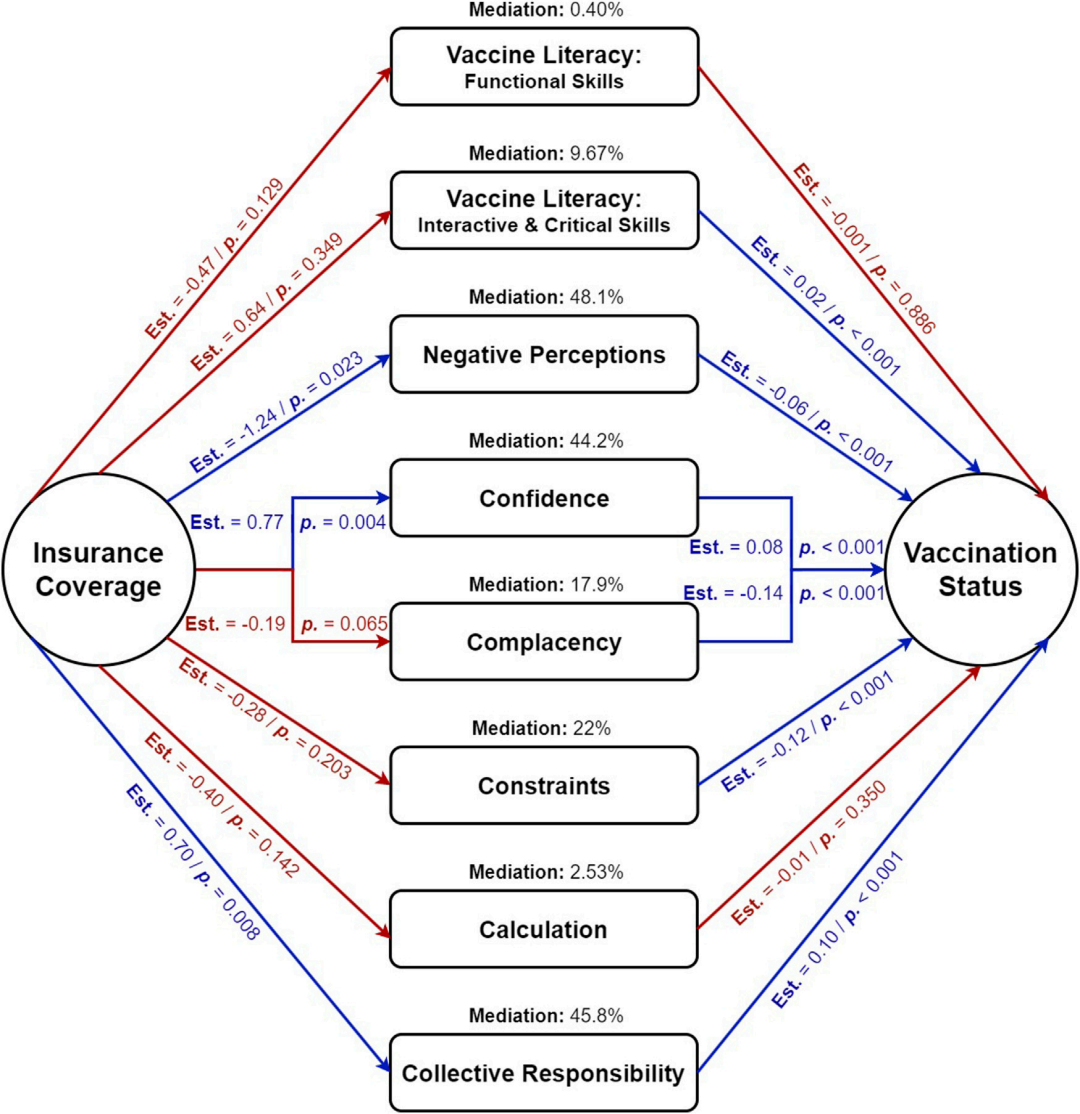
Mediation analysis of psychological and behavioral factors mediating the impact of insurance coverage (predictor) on seasonal influenza vaccination status (outcome) among senior Czechs responding to the Influenza Vaccination Survey, Czech Republic, April–August 2023 (*n* = 384).

Negative perceptions (48.1%), confidence (44.2%), and collective responsibility (45.8%) were the most substantial mediators. Reducing negative perceptions, enhancing vaccine confidence, and fostering community responsibility can significantly impact vaccination status among insured individuals. Other mediators included constraints (22%), complacency (17.9%), and interactive and critical skills (9.7%), highlighting the need to improve vaccine literacy and address perceived barriers. Functional skills (0.4%) and calculation (2.5%) were less significant, indicating a minor influence on the relationship between insurance coverage and vaccination status (Figure 1).

## Discusssion

This study assessed seasonal influenza vaccine literacy and hesitancy among elderly Czechs using the 5C model of psychological antecedents. Key findings showed that higher interactive and critical vaccine literacy, lower negative perceptions, greater vaccine confidence, and a stronger sense of collective responsibility were significantly linked to increased vaccination rates. Insurance coverage was the most significant sociodemographic factor. The results highlight the importance of lowering the insurance coverage age and addressing confidence, collective responsibility, and negative perceptions to boost vaccination uptake among the elderly.

### Impact of Vaccine Literacy on Hesitancy

The concept of vaccine literacy, historically adapted from health literacy, shares common features with its progenitor, including its key components: functional, interactive, and critical literacy [10]. A concept analysis of Badua et al. emphasized the integration of vaccine literacy into broader health literacy strategies, underscoring the need for communication and engagement to combat vaccine hesitancy among seniors [10]. Additionally, Michel et al. found that effective, tailored vaccine communication enhances vaccine uptake among the elderly. Healthcare professionals, as key knowledge brokers, require specialized training to effectively convey information to this demographic [13].

A deeper examination of key constructs of vaccine literacy—functional skills and interactive-critical skills—reveals a heterogeneous influence of each construct on vaccination intentions and behaviors [11, 12]. In our study, critical skills were significantly associated with vaccine uptake (*p.* < 0.001), whereas functional skills showed no significant association (*p.* = 0.664). Moreover, the impact of insurance coverage on seasonal influenza vaccine status was mediated by critical skills at 9.7% compared to functional skills at only 0.4%. In line with our findings, a Tunisian study found that among cancer patients, interactive-critical skills strongly correlated with COVID-19 vaccine acceptance, while functional skills showed no association with vaccination willingness [35]. Also, among patients with systemic autoimmune diseases, interactive-critical skills were linked to positive beliefs about the COVID-19 vaccine [36]. In Iran, a cross-sectional study among adults found that critical skills were significantly associated with COVID-19 vaccine acceptance, while functional skills were not [37].

Research on influenza vaccine literacy further supports this distinction. Shon et al. found that flu vaccine literacy was a key predictor of positive health beliefs and higher flu vaccination rates among undergraduate students in Ohio [38]. A recent Chinese study investigated the relationship between influenza vaccine hesitancy and vaccine literacy among young adults; and it found out that higher competence and decision-making literacy were strongly associated with influenza vaccination behaviour, whereas knowledge literacy was paradoxically linked to lower vaccination uptake [39]. After a 2016 vaccine scandal in China involving improperly stored vaccines, Wang et al. studied parental trust and vaccine acceptance. They found that critical vaccine literacy (OR = 3.49) significantly influenced vaccine acceptance more so than functional literacy (OR = 1.81) [40]. The recent review by Biasio et al. on current vaccine literacy tools underscores the need for new tools that integrate knowledge, competencies, and psychological components related to motivation [14]. The review highlights the effectiveness of using current items of the interactive-critical construct for assessing motivation and competencies in understanding and evaluating vaccination information [14].

### Confidence and Collective Responsibility as Vaccination Promoters

Our study indicated that confidence (AOR = 1.60) and collective responsibility (AOR = 1.80) were significant promoters of seasonal influenza vaccine uptake. Likewise, studies utilizing the 5 C model have found out that confidence and collective responsibility were significantly associated with higher odds of COVID-19 vaccine acceptance among various population groups, e.g., Black Americans [41], US veterans [42], Saudi parents [43], adults in thirteen Arab countries [44], Indian adults [18], adults in Zambia, Nepal, and Senegal [45], healthcare workers in Sudan [46], as well as university students in the Netherlands, Belgium, and Portugal [16]. In Japan, a longitudinal study aimed to identify trends in COVID-19 vaccination intent revealed that it increased post-vaccine distribution, with confidence and collective responsibility positively influencing acceptance, while calculation negatively affected intent across all age and sex groups [47]. In a different context, acceptance of the monkeypox vaccine among Ghanaian population was significantly associated with higher confidence (AOR = 2.45) and collective responsibility (AOR = 1.34) [48].

Vaccine acceptance is influenced by scientific, psychological, sociocultural, and political factors. Public concerns extend beyond safety to include policies, costs, and new research findings. Effective communication must be context-specific, transparent, and address public concerns to build trust in vaccines [49]. In our study, while confidence was significantly associated with insurance coverage, chronic diseases, and COVID-19 and pneumococcal vaccination, it was not influenced by sex, education level, or income level. Contrarily, sex had a significant impact on confidence among Japanese adults (females > males) [47], Arab adults in thirteen countries (females > males) [44], and Saudi parents (males > females) [43]. Higher educational and income levels were significantly associated with higher confidence in several studies [44, 50].

### Lowering Recommended Age Cutoff

Lowering the recommended age for influenza vaccination has long been advocated by health experts. In 1999, the American Academy of Family Physicians (AAFP) recommended annual influenza vaccination for individuals aged 50 and older, citing significant reductions in morbidity, hospitalizations, and associated healthcare costs [20, 51]. In Italy, a study aimed to estimate the clinical and economic impact of lowering the recommended age for influenza vaccination to 50 years in the Liguria region found out that this policy could reduce annual influenza cases by up to 13.8%, emergency department visits by up to 15.4%, complications by up to 14.7%, and hospitalizations by up to 15.4%, demonstrating both health benefits and potential cost savings for the healthcare system [21]. Additionally, a Spanish study compared regions that lowered the age limit to 60 years with those that maintained it at 65 years, and found that regions with the lowered age limit had significantly higher vaccination rates across all age groups, particularly among individuals aged 60 to 64 without chronic diseases (36.9% vs. 24.4%) [22].

Nevertheless, the current practice in the Czech Republic is that influenza vaccination is only recommended for adults aged 65 and older, and for adults with certain chronic diseases regardless of age, such as chronic cardiovascular disease, chronic kidney disease, and diabetes mellitus. Consequently, the national health insurance scheme fully covers the vaccine costs only for these two population groups, as stipulated by Act No. 48/1997 [19]. In 2022, the Czech Vaccinology Society (CVS) recommended annual influenza vaccination for all individuals from 6 months of age, with a specific emphasis on high-risk groups such as seniors, young children, pregnant women, and those with chronic diseases [52]. Notably, the CVS highlighted a shift in the age recommendation for seniors, advocating vaccination starting at 50 years of age instead of the current threshold of 65 [52].

In Europe, there are significant variations in the recommended age for influenza vaccination. Some countries have universal vaccination recommendations (Austria, Estonia, and Poland), while others have lower cutoffs: 50 years (Belgium and Ireland), 55 years (Malta), 59 years (Slovakia), and 60 years (Germany, Greece, Iceland, the Netherlands, and Portugal) [5]. Lowering the vaccination age below 65 could increase coverage and reduce influenza morbidity and healthcare costs. However, this policy might be seen as excessive since immunosenescence generally starts between 65 and 70 years [53].

In our study, interactive and critical skills of vaccine literacy (9.7%), confidence (44.2%), collective responsibility (45.8%), and negative perceptions (48.1%) significantly mediated the impact of insurance coverage, as indicated by the age limit (65 years), on seasonal influenza vaccine uptake. These results suggest that if the Czech Republic decides to lower the recommended age for vaccination from 65 to 55, this policy should be accompanied by additional measures to improve vaccine literacy, enhance public confidence in vaccines, and foster a sense of collective responsibility. Efforts to combat misinformation and address negative perceptions are also critical. By implementing these multifaceted interventions, we can ensure a satisfactory return on investment for any economic measures taken to lower the recommended age for influenza vaccination in the near future.

### Limitations

This study has several limitations. The distribution of the SAQ through Universities of the Third Age and community organizations primarily targeted socially active seniors, potentially leading to social selection bias. Despite efforts to consider computer literacy, the digital dissemination may have excluded less tech-savvy individuals. Additionally, attempts to reach more isolated seniors in senior homes and assisted living facilities were unsuccessful, limiting the generalizability of the findings. The reliance on a quantitative design with the SAQ did not capture the depth of respondents’ specific views and experiences. Finally, using a modified short version of the 5-C scale with 12 items instead of 15 items may limit comparability of our findings to other studies using this scale.

### Implications

Our findings highlight that while lowering the age of insurance coverage for seasonal influenza below 65 in the Czech Republic may be beneficial, it must be accompanied by targeted efforts to address mediating factors such as confidence, collective responsibility, and negative perceptions. To ensure the policy’s success, it is essential to implement educational campaigns that enhance vaccine literacy, boost vaccine confidence, and foster a sense of collective responsibility. Additionally, addressing negative perceptions and combating misinformation is crucial for the policy’s success and to maximize the return on investment of any economic measures taken.

Moreover, we found that most participants relied on their general practitioners for influenza vaccination. Therefore, integrating vaccine literacy training for healthcare providers could enhance their ability to effectively address patient concerns, particularly regarding vaccine safety and effectiveness.

### Conclusion

In conclusion, this study emphasizes the need to enhance vaccine literacy and address psychological factors to boost influenza vaccination rates among the elderly. Higher vaccine literacy, reduced negative perceptions, increased confidence, and a sense of collective responsibility were linked to greater vaccination uptake. To maximize the benefits of lowering the vaccination age cutoff, it is crucial to also improve vaccine literacy, build public trust, and counter negative perceptions. These measures are vital to ensure the policy’s success and achieve better health outcomes for the elderly.

## Data Availability

The data that support the findings of this study are available from the corresponding author upon reasonable request.
