# Supplementary material for: Seasonal Influenza Vaccine Literacy and Hesitancy of Elderly Czechs: An Analysis Using the 5C Model of Psychological Antecedents
Source: Int J Public Health. 2024 Oct 14;69:1607626. doi: 10.3389/ijph.2024.1607626 (PMC11513313; doi:10.3389/ijph.2024.1607626)
Supplement: Supplementary file 1 [file Table1.DOCX]

**Table S1.** Sociodemographic and Anamnestic Characteristics of Senior Czechs Responding to Influenza Vaccination Survey, April – August 2023, (*n* = 384)

| **Variable** | **Outcome** | **Never Vaccinated**  **(*n* = 145)** | **Ever Vaccinated**  **(*n* = 239)** | **Total**  **(*n* = 384)** | ***Sig.*** |
| --- | --- | --- | --- | --- | --- |
| **Sex** | Female | 118 (81.9%) | 186 (77.8%) | 304 (79.4%) | 0.334 |
|  | Male | 26 (18.1%) | 53 (22.2%) | 79 (20.6%) |  |
| **Age** | Median (IQR) | 66 (61 – 71.5) | 70 (64 – 75) | 68.5 (62 – 74) | **<0.001** |
| **Coverage** | No (< 65 years) | 59 (40.7%) | 65 (27.2%) | 124 (32.3%) | **0.006** |
|  | Yes (≥ 65 years) | 86 (59.3%) | 174 (72.8%) | 260 (67.7%) |  |
| **Education** | Elementary | 2 (1.4%) | 4 (1.7%) | 6 (1.6%) | 0.973 |
|  | Secondary | 81 (55.9%) | 131 (54.8%) | 212 (55.2%) |  |
|  | University | 62 (42.8%) | 104 (43.5%) | 166 (43.2%) |  |
| **Income** | Pension Only | 88 (60.7%) | 143 (59.8%) | 231 (60.2%) | 0.868 |
|  | Pension + Additional Sources | 57 (39.3%) | 96 (40.2%) | 153 (39.8%) |  |
| **Residence** | Own household | 142 (97.9%) | 238 (99.6%) | 380 (99%) | 0.244 |
|  | Elderly Home | 2 (1.4%) | 1 (0.4%) | 3 (0.8%) |  |
|  | Nursing Home | 1 (0.7%) | 0 (0%) | 1 (0.3%) |  |
| **Smoking** | No | 126 (86.9%) | 211 (88.3%) | 337 (87.8%) | 0.687 |
|  | Yes | 19 (13.1%) | 28 (11.7%) | 47 (12.2%) |  |
| **BMI Level** | Normal | 49 (34.5%) | 76 (32.1%) | 125 (33%) | 0.953 |
|  | Overweight | 61 (43%) | 103 (43.5%) | 146 (43.3%) |  |
|  | Obese | 21 (14.8%) | 37 (15.6%) | 58 (15.3%) |  |
|  | Extremely Obese | 11 (7.7%) | 21 (8.9%) | 32 (8.4%) |  |
| **Chronic Diseases** | Overall | 50 (34.5%) | 119 (49.8%) | 169 (44%) | **0.003** |
|  | Allergy | 15 (30%) | 30 (25.2%) | 45 (26.6%) | 0.520 |
|  | Asthma | 12 (24%) | 20 (16.8%) | 32 (18.9%) | 0.276 |
|  | Blood Disease | 2 (4%) | 5 (4.2%) | 7 (4.1%) | 1.000 |
|  | Bone Disease | 2 (4%) | 1 (0.8%) | 3 (1.8%) | 0.209 |
|  | GIT Disease | 3 (6%) | 10 (8.4%) | 13 (7.7%) | 0.757 |
|  | Cancer | 3 (6%) | 3 (2.5%) | 6 (3.6%) | 0.362 |
|  | Cardiovascular Disease | 6 (12%) | 30 (25.2%) | 36 (21.3%) | 0.056 |
|  | Chronic Hypertension | 20 (40%) | 62 (52.1%) | 82 (48.5%) | 0.151 |
|  | COPD | 3 (6%) | 4 (3.4%) | 7 (4.1%) | 0.423 |
|  | Diabetes Mellitus I | 2 (4%) | 1 (0.8%) | 3 (1.8%) | 0.209 |
|  | Diabetes Mellitus II | 11 (22%) | 22 (18.5%) | 33 (19.5%) | 0.599 |
|  | Hepatologic Disease | 0 (0%) | 2 (1.7%) | 2 (1.2%) | 1.000 |
|  | Psychologic Disorder | 4 (8%) | 9 (7.6%) | 13 (7.7%) | 1.000 |
|  | Neurologic Disorder | 8 (16%) | 16 (13.4%) | 24 (14.2%) | 0.664 |
|  | Ophthalmic Disease | 4 (8%) | 8 (6.7%) | 12 (7.1%) | 0.750 |
|  | Renal Disease | 1 (2%) | 1 (0.8%) | 2 (1.2%) | 0.505 |
|  | Rheumatoid Arthritis | 3 (6%) | 10 (8.4%) | 13 (7.7%) | 0.757 |
|  | Thyroid Disorder | 14 (28%) | 31 (26.1%) | 45 (26.6%) | 0.794 |
| **Medications** | Overall | 109 (75.2%) | 201 (84.1%) | 310 (80.7%) | **0.032** |
|  | Antiasthma | 9 (8.3%) | 24 (11.9%) | 33 (10.6%) | 0.315 |
|  | Anticoagulants | 6 (5.5%) | 22 (10.9%) | 28 (9%) | 0.111 |
|  | Antidepressive | 9 (8.3%) | 24 (11.9%) | 33 (10.6%) | 0.315 |
|  | Antidiabetics | 17 (15.6%) | 28 (13.9%) | 45 (14.5%) | 0.691 |
|  | Anti-epileptics | 2 (1.8%) | 0 (0%) | 2 (0.6%) | 0.123 |
|  | Antihistamines | 7 (6.4%) | 18 (9%) | 25 (8.1%) | 0.434 |
|  | Antihypertensive | 48 (44%) | 109 (54.2%) | 157 (50.6%) | 0.087 |
|  | Anti-reflux | 8 (7.3%) | 17 (8.5%) | 25 (8.1%) | 0.730 |
|  | Immunosuppressive | 1 (0.9%) | 2 (1%) | 3 (1%) | 1.000 |
|  | Cholesterol-lowering | 50 (45.9%) | 100 (49.8%) | 150 (48.4%) | 0.514 |
|  | Common Analgesics | 5 (4.6%) | 19 (9.5%) | 24 (7.7%) | 0.126 |
|  | Corticosteroids | 2 (1.8%) | 8 (4%) | 10 (3.2%) | 0.503 |
|  | NSAIDs | 2 (1.8%) | 6 (3%) | 8 (2.6%) | 0.717 |
|  | Opioids | 0 (0%) | 2 (1%) | 2 (0.6%) | 0.543 |
|  | Thyroid Hormones | 27 (24.8%) | 50 (24.9%) | 77 (24.8%) | 0.984 |
| **COVID-19 Vaccine** | No | 17 (11.7%) | 9 (3.8%) | 26 (6.8%) | **0.003** |
|  | Yes | 128 (88.3%) | 230 (96.2%) | 358 (93.2%) |  |
| **COVID-19 Vaccine Doses** | Primer Doses Only | 24 (18.8%) | 15 (6.5%) | 39 (10.9%) | **<0.001** |
|  | Primer + 1 Booster | 38 (29.7%) | 65 (28.3%) | 103 (28.8%) |  |
|  | Primer + 2 Boosters | 66 (51.6%) | 150 (65.2%) | 216 (60.3%) |  |
| **Pneumococcal Vaccine** | No | 122 (84.1%) | 158 (66.1%) | 280 (72.9%) | **<0.001** |
|  | Yes | 23 (15.9%) | 81 (33.9%) | 104 (27.1%) |  |

Chi-squared test (*χ^2^*), Fisher’s exact test, and Mann-Whitney test (*U*) were used with a significance level < 0.05.

**Table S2.** Influenza Vaccination-related Anamnesis as Reported by Senior Czechs, April – August 2023, (*n* = 384)

| **Variable** | **Outcome** | **Frequency (*n*)** | **Percentage (*%*)** | **Median (IQR)** |
| --- | --- | --- | --- | --- |
| **Influenza Vaccination** | No | 145 | 37.8 |  |
|  | Yes | 239 | 62.2 |  |
| **Latest Dose** | Less than 12 months | 173 | 72.4 |  |
|  | Less than 24 months | 19 | 7.9 |  |
|  | Less than 36 months | 9 | 3.8 |  |
|  | More than 36 months | 38 | 15.9 |  |
| **Provider** | General Practitioner | 200 | 83.7 |  |
|  | Vaccination Centre | 38 | 15.9 |  |
|  | Social/Healthcare Staff | 1 | 0.4 |  |
| **Infected** | No | 213 | 89.1 |  |
|  | Yes | 26 | 10.9 |  |
| **Infection Severity** | VAS |  | | 6 (3.75 – 8) |
